# General Practitioner Use of e-Consultation to Consult Hospital Specialists: Interview Study to Obtain Physician’s Perceptions About Digital Interprofessional Communication

**DOI:** 10.2196/40318

**Published:** 2023-03-28

**Authors:** Ken Peeters, Esther Giroldi, Lotte Veldhuizen, Kahin Abdelaziz, Dennis Muris, Jochen Cals

**Affiliations:** 1 Department of Family Medicine Care and Public Health Research Institute Maastricht University Maastricht Netherlands; 2 Zuyderland Medical Centre Sittard Netherlands; 3 Medical Coordination Centre Omnes Sittard Netherlands; 4 Department Educational Development & Research School of Health Professions Education Maastricht University Maastricht Netherlands

**Keywords:** e-consultation, e-consult, electronic consultation

## Abstract

**Background:**

In recent years, tasks have shifted from specialized hospital care to primary care, leading to both advantages and challenges for general practitioners (GPs). A frequently mentioned tool to face these challenges is e-consultation, a form of asynchronous digital interprofessional communication between GPs and hospital specialists.

**Objective:**

The objective of this study was to gain insight into GPs’ and hospital specialists’ views and experiences of e-consultation.

**Methods:**

We interviewed 47% (15/32) GPs and 53% (17/32) hospital specialists and performed a thematic analysis.

**Results:**

We found that both GPs and hospital specialists experience a positive effect on the quality of care and collaboration between GPs and hospital specialists. Positive effects were reported on the accessibility of care, efficiency of care, and relationship between the GP and the patient. Furthermore, communication between GP and hospital specialist became more efficient, and e-consultation offered educational value for the GP. Certain improvements are needed to further optimize e-consultation, regarding applicability, communication, and training purposes.

**Conclusions:**

In the future, clinicians and policy makers can use the insights gained from this study to further optimize and implement e-consultation in clinical practice.

## Introduction

### Background

In recent years, there has been an increasing demand for primary care services in the Netherlands. A frequently mentioned change is the shift from specialized hospital care to primary care, also known as substitution of care [1]. Shifting tasks from specialized hospital care to primary care can lead to many advantages in terms of quality of care, short waiting lists, low health care costs, low costs for patients, and few hospital referrals [2,3]. General practitioners (GPs) have a crucial role as gatekeepers in the Dutch health care system. Substitution of care poses many difficulties and challenges for GPs, such as increasing workload and work stress. Moreover, the increasing part-time positions of physicians both in primary and secondary care create additional challenges for direct communication regarding patient-related questions [4-10]. In addition, GPs often encounter diagnostic uncertainty or gaps in their knowledge during patient care. A frequently mentioned tool to address these challenges is e-consultation [3], a form of asynchronous electronic communication between GPs and hospital specialists. e-Consultation is initiated through a secured digital platform in which GPs disclose a specific clinical question to a hospital specialist or seek advice about the care of a particular patient, after which the specialist submits an answer to the GP [11,12]. e-Consultation is unidirectional, used for patients who are not yet under treatment by a hospital specialist, and the communication between the GP and specialist is stored in a shared and secured electronic record. We believe that e-consultation may increase the quality of care, which is defined by the World Health Organization as “the degree to which health services for individuals and populations increase the likelihood of desired health outcomes” [13]. There is growing acknowledgment that quality health services should account to a number of criteria, which are effectiveness, safety, people-centeredness, timeliness, equity, integration, and efficiency [13].

e-Consultations may offer major advantages for physicians in comparison with other consultations [11,14,15]. For instance, in a previous study, several physicians have stated that e-consultations cause less disruptions in clinical work compared with informal consultations, as they can be initiated or responded to when they have available time [11]. It also allows physicians to share patient information, such as recent diagnostic results or medical history easily and securely. In addition, the communication (and shared advice) between both physicians is documented immediately [14]. Although these studies identified many positive elements of e-consultations, it appears that there is still scope for improvements, such as those described previously. Insufficient information in the stated question by GPs, resulting in unintended telephone or face-to-face appointments between the hospital specialist and patient, further add to the workload [4,16,17].

To improve e-consultation further and implement it on a wide scale in the region and across nonsurgical and surgical specialties, it is crucial to thoroughly understand the experiences and views of the end users and to explore if similar or contrasting themes emerge in the perceptions of GPs and hospital specialists.

### Objective

In this qualitative study, we interviewed GPs and hospital specialists about their experiences and perspectives regarding e-consultation, allowing us to understand the impact of e-consultation on patient care. Furthermore, these insights help us to further optimize e-consultation in its current state.

## Methods

### Research Design

We conducted a qualitative study to explore the views of GPs and hospital specialists on e-consultation. The research team conducted semistructured interviews and analyzed them using inductive thematic analysis. The study adheres to the principles for reporting qualitative research [18].

### Ethics Approval

The ethical committee of Zuyderland-Zuyd (protocol number 14-N-69) approved this study.

### e-Consultation Process

**Figure 1:**
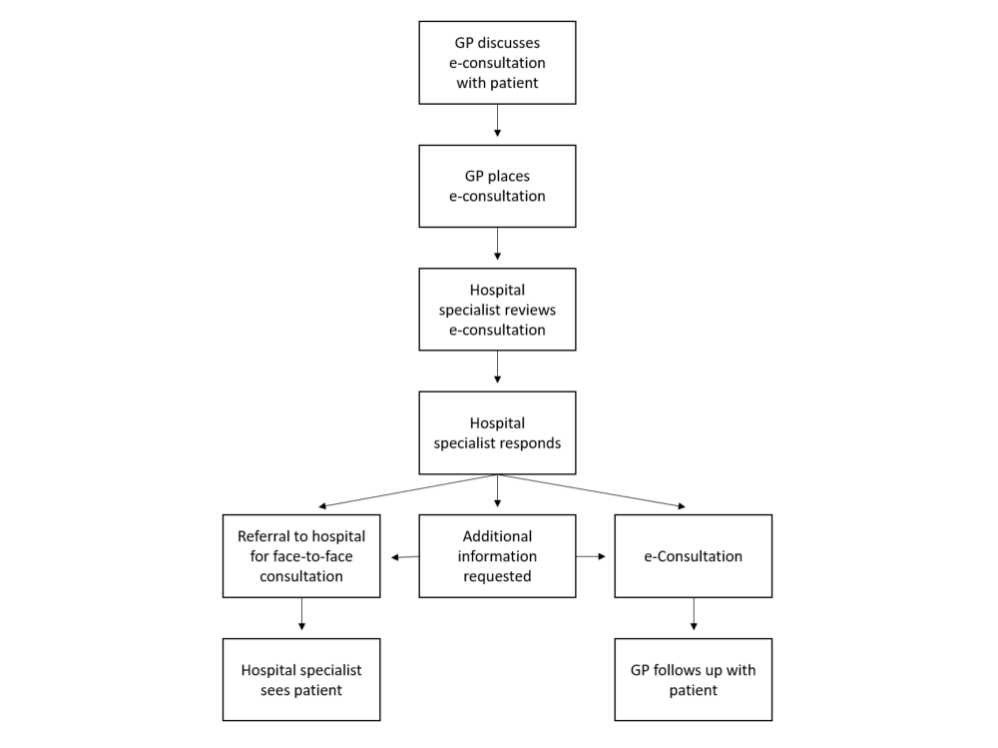
e-Consultation process. GP: general practitioner.

Currently, e-consultations are available for GPs located in the south of the Netherlands. In this region, GPs can submit e-consultations to hospital specialists of participating specialties in the hospital in the region. Figure 1 illustrates the process of an e-consultation. Before submitting an e-consultation, the GP informs the patient. Subsequently, the GP submits the e-consultation through secured referral application, *ZorgDomein* (ZorgDomein). In the e-consultation, the GP discloses a specific clinical question about a patient, along with further patient information, such as medical history and recent diagnostic results. An assistant schedules the e-consultation in the agenda of the hospital specialist, who answers and returns this to the GP through an electronic letter via Edifact within 2 business days. Follow-up questions are not possible. In addition, an e-consultation is not possible when a patient is already under specialist treatment for that medical condition [12].

### Study Population and Setting

We purposely invited and selected GPs from 1 geographically demarcated region in the Netherlands collaborating with a large regional hospital (Zuyderland Medical Centre, Sittard-Geleen) [19] that requested at least one e-consultation between January 1, 2017, and December 31, 2019. This is the period during which e-consultation was implemented for different departments. All GPs continued using e-consultation thereafter. We purposely included GPs with a broad range of experience in sending e-consultations. Furthermore, we purposely only included hospital specialists who regularly answered e-consultations for their departments. We specifically selected hospital specialists from the departments of internal medicine, gastroenterology, orthopedics, surgery, pulmonology, and cardiology, as we aimed to obtain experiences and perceptions from hospital specialists from the specialties with a substantial number of e-consultations per month and a range of surgical and nonsurgical disciplines. Specialists were financially compensated for answering e-consultations; however, the compensation was smaller than that of a face-to-face visit. Assistants scheduled time for answering e-consultations in the specialist’s agenda. We acquired the contact information of GPs and hospital specialists through the organization, MCC Omnes, which had the permission to enclose such information for research purposes, and recruited participants through either email or direct call. We strived to include 15 to 20 interviews per group, as estimates by Moser et al [20] have shown that content analysis requires 15 to 20 interviews.

### Data Collection

We conducted semistructured interviews with participants from December 2020 to April 2021. The research team developed a topic list (Multimedia Appendix 1), which was tested via a pilot interview to ensure that the questions were constructed in an understandable manner. In this study, 2 female student researchers with previous training in qualitative research (LV and KA, MSc) conducted all semistructured interviews. The interviews focused on the experience of GPs and hospital specialists with e-consultations, attitude of GPs and hospital specialists toward e-consultation, impact of e-consultation on patient care and relationships, and access to specialty care. Researchers had no previous relationship with participants, and participants had no knowledge about the goals or characteristics of the researchers. We used open-ended questions to facilitate the discussion and acquire more in-depth answers. In summary, we asked participants about their general thoughts about e-consultation, applicability of e-consultation within their own department, general benefits, and recommendations for improvement. Interviews lasted approximately 30 minutes. No repeat interviews were conducted. Owing to the COVID-19 pandemic during the research period, one-on-one interviews with GPs and hospital specialists were conducted through a secured digital platform, such as Zoom Videoconferencing or Microsoft Teams, while interviewers made field notes. After obtaining informed consent, we audio-recorded and transcribed the interviews verbatim. During transcription, we removed all identifiable information and anonymized the data as much as possible. Data were collected and analyzed using an iterative process, to improve the quality of subsequent interviews.

### Data Analysis

The team used NVivo (version 11; QSR International), a qualitative data management software, for data analysis. We read transcripts thoroughly and coded them using inductive and open coding, meaning that we did not use any preconceived codes and codes evolved and changed over time. We selected segments of text and labeled them with a code that described the content. One researcher (LV) coded most of the GP interviews, whereas another researcher (KA) coded most of the hospital specialist interviews. To enhance trustworthiness, each researcher also coded 25% of the transcripts from the other group, meaning that 25% of the transcripts were double coded. Researchers discussed both codes and compared them until they reached consensus. During the analysis process, the research team identified common overarching themes and underlying subthemes. Themes were continually refined during analysis. As a member check, we sent an overview of the results to all participants, which did not lead to any changes in the analysis. Researchers conducted interviews until data saturation was reached; that is, no new and relevant themes were raised.

## Results

### Overview

**Figure 2:**
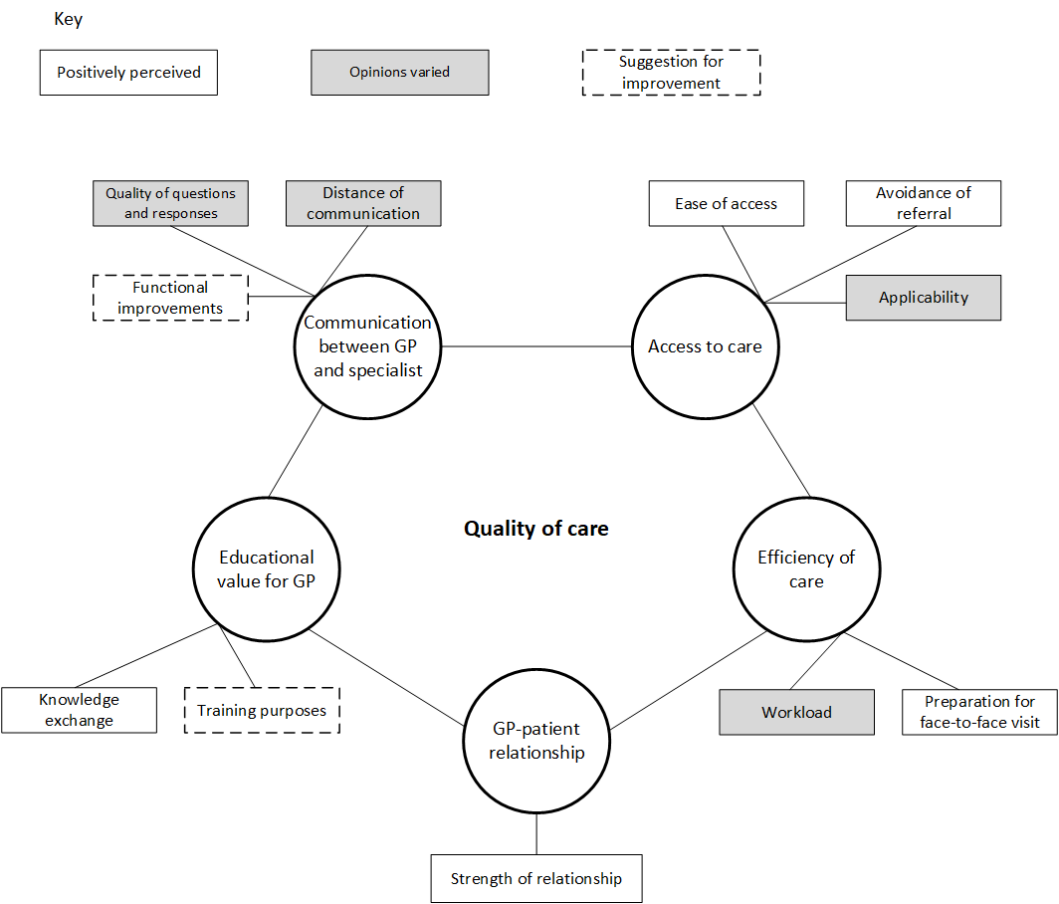
Themes that are influenced by the use of e-consultations, as identified in the interviews. GP: general practitioner.

We performed interviews with 47% (15/32) GPs and 53% (17/32) hospital specialists before reaching data saturation. There were no dropouts. Table 1 presents the characteristics of the interviewed participants. Thematic analysis of the interviews identified 5 major themes and related subthemes (Figure 2). Each of the themes is described in the following sections. Each theme is illustrated using quotations (Table 2).

**Table 1:** Baseline characteristics of participants.

			Participants (N=32), n (%)		Age (years), mean (SD)		Experience (years), mean (SD)		Sex (male), n (%)		Group practice, n (%)		Answered e-consultations^a^, mean (SD)		e-Consultations received^b^ (N=952), n (%)
General practitioners			15 (47)		47.5 (9.9)		15.3 (8.9)		9 (60)		14 (93)		N/A^c^		N/A
**Hospital specialists**			17 (53)		44.2 (7.8)		11.4 (6.7)		13 (75)		N/A		12.9 (9.7)		476 (50)
	Internal medicine	5 (29)		39.5 (8.1)		9 (6.1)		1 (25)		N/A		26 (7.4)		234 (24.6)	
	Gastroenterology	2 (12)		49 (1)		13.5 (0.5)		1 (50)		N/A		7.2 (2.5)		88 (9.2)	
	Cardiology	2 (12)		52.5 (8.5)		18 (7)		1 (50)		N/A		12 (4)		77 (8.1)	
	Pulmonology	2 (12)		48.5 (6.5)		15.5 (8.5)		2 (100)		N/A		8 (0)		31 (3.3)	
	Surgery	4 (24)		44.3 (3.3)		8.8 (3.6)		4 (100)		N/A		4.9 (2.9)		19 (1.9)	
	Orthopedics	2 (12)		36 (0)		9 (6)		1 (50)		N/A		11.8 (5.8)		27 (2.8)	

^a^As stated by hospital specialist; per month.

^b^e-Consultations received by the departments of the interviewed hospital specialists during the fourth quarter of 2020—the period before the interviews were conducted.

^c^N/A: not applicable.

**Table 2:** Themes, subthemes, and quotations.

Themes and subthemes			Quotations
**Reasons to request**			
	N/A^a^	1. “I see it as a GP^b^ ‘borrowing’ our brain, while still treating the patient themselves.” [SP^c^10] 2. “It depends on the nature of the specialty. If a patient needs an intervention, it is unnecessary to keep the patient in the first line. A referral to us surgeons is required anyway.” [SP5]	
**Communication**			
	Asynchronous nature of communication	3. “To be able to share diagnostics is an advantage. The specialist is able to view all information.” [GP1] 4. “It’s unfortunate that we don’t have face-to-face contact anymore or even talk on the phone, which normally strengthens the interpersonal relationship.” [SP4]	
	Quality of questions and responses	5. “I want additional information. I don’t just want an advice, for example ‘I’d give this medication etc.’ I want to know why and how.” [GP10] 6. “It’s difficult evaluating a consultation when there is limited information. I don’t want to miss or misinterpret an important diagnosis.” [SP14]	
	Functional improvements	7. “I think it would benefit the quality of the consultation if the specialist also receives feedback on his answer. Not necessarily about the medical contents, but the usability for the GP.” [SP7] 8. “I don’t see the necessity for that. I personally think it is better to discuss something over the phone if something is unclear, or just to refer the patient. A feedback or chat function would exceed the goal of the e-consultation and just take a lot of extra time.” [SP2]	
**Access to care**			
	Ease of access	9. “I think that because of an e-consultation, the barrier to consult a specialist is very low for a GP.” [SP10] 10. “I like the speed. They have set a term of 48 hours, which means you can quickly inform your patient again. Patients greatly appreciate this. They also appreciate not having to make a trip to the hospital. Especially during these times [COVID-19] in which people are afraid to go to the hospital.” [GP14]	
	Avoidance of referral	11. “Some patients want the reassurance of a specialist. As a GP you often don’t see the need for this referral. In the past, you would still refer these patients but because of the e-consultation, this is not necessary anymore.” [GP3]	
	Applicability	12. “I do feel that the GP is still searching for the correct situations in which an e-consultation is necessary.” [SP17]	
**Efficiency of care**			
	Preparation	13. “It also gives us an opportunity to request the GP to already do certain tasks [for example diagnostics] before referring the patient to us.” [SP15]	
	Workload	14. “Normally you have to contact all kinds of intermediaries to reach a specialist, which takes time and proposes a dilemma for a GP because you want to get back to the patient quickly. So I actually think that because of an e-consultation the workload decreases.” [GP5] 15. “I have to send them the consultation, wait for an answer, actively look at it, decide on which actions to take and call the patient. So it is a little bit more work.” [GP2]	
**Educational value**			
	Knowledge exchange	16. “The educational value is much greater. You get a response quite fast and the majority of the specialists also substantiate their advice. That is very helpful. I use it a lot, and even made maps, in which I stored frequently asked questions and responses.” [GP14] 17. “Ideally, I would hope we also train the GP’s with these e-consults. In the beginning they might need more of them, but if they have received the same answer multiple times they will be able to handle these types of questions themselves.” [SP6]	
	Training purposes	18. “I would suggest to collect frequently asked questions and use these for training purposes of GP’s, that would complete the cycle of learning and be really interesting.” [GP7]	
**GP-patient relationship**			
	Strength of relationship	19. “I do believe that patients trust us more when our advice is backed by a specialist, so that improves our relationship with the patient.” [GP12]	

^a^N/A: not applicable.

^b^GP: general practitioner.

^c^SP: hospital specialist.

### e-Consultation—Why and When?

The expressed appreciation to be able to consult a hospital specialist in secondary care was enormous. GPs acknowledge experiencing a gap in knowledge or lack of expertise when asked about certain questions by patients. GPs stated that direct (telephone) communication to deliberate on patient-related questions was preferred. The inaccessibility and long wait times for hospital specialists during telephone consultations make GPs reserved about consulting a hospital specialist via traditional telephone communication. Both GPs and hospital specialists expressed that the increase in the number of part-time physicians made direct contact more challenging from both sides nowadays.

Reported reasons to request an e-consultation for GPs are the following: staying in control of direct patient care, avoiding unnecessary referrals when experiencing a gap in specific knowledge, and providing patients with an alternative upon a patient-initiated request for referral to secondary care for reassurance. GPs expressed that e-consultation makes access to specialist knowledge and consultation easy, and some GPs noted that the threshold to ask a question is low compared with that of telephonic consultation (quote 1).

Both GPs and hospital specialists express that nonsurgical specialisms such as internal medicine and cardiology are more suited for e-consultation compared with surgical specialisms such as orthopedics and general surgery. Reported reasons for this are that questions directed to surgical specialisms are more specific compared with nonsurgical specialisms and provide less room for interpretation. Furthermore, surgical specialists report that, often, they need to see the patient in person to physically evaluate them (eg, wounds). Finally, some surgical specialists report that patients sent to them often require an intervention; therefore, a face-to-face referral is required anyway (quote 2).

### Communication Between GP and Hospital Specialist

#### Asynchronous Nature of Communication

Both GPs and hospital specialists agreed that owing to the asynchronous nature of e-consultation, communication has become more efficient. The ability to take time to formulate a good clinical question, provided with relevant clinical information including findings from GP-initiated diagnostic testing, makes communication of the problem clear and more efficient. Furthermore, the patient file shared between primary and secondary care makes consultation more efficient and complete and has the advantage that both the e-consultation itself and the answer provided by hospital specialists are registered in the general practice medical record and hospital record, in contrast to advice being asked or provided via telephone (quote 3). GPs and hospital specialists reported on a decrease in telephone consultation with specialists and therefore less disruption of work. After implementation of e-consultation, GPs and hospital specialists primarily use telephonic consultations in need of emergency or when discussing a patient already under specialist treatment. However, GPs see the importance of always having the opportunity to call a specialist in specific urgent cases. By having this opportunity, GPs can call a specialist of their choice and provide them with more context. Some GPs reported on being afraid that specialists will hold off telephone consultations when e-consultation is available.

Despite the perceived positive effects on GP–hospital specialist communication, GPs and hospital specialists also regret the fact that interprofessional contact has become more distant and anonymous, which makes it more difficult to build on the personal GP–hospital specialist relationship (quote 4). Communication is more impersonal owing to the unidirectional nature. Furthermore, a few hospital specialists revealed that they found it challenging to express their opinions and share their thoughts through a nonverbal communication platform. They were unsure whether GPs interpret their message or advice correctly and experienced some loss of control.

#### Quality of Questions and Responses

Both parties agreed that a useful e-consultation consists of a high-quality question and response, which requires dedication from both GP and hospital specialist to effectively query and respond, respectively. GPs appreciate hospital specialists offering clear and elaborate diagnostic and therapeutic guidelines for different possible situations (quote 5). GPs do regret that the quality of responses differ between individual hospital specialists. However, they acknowledged that poor-quality responses might be a reaction to unclear clinical questions. Hospital specialists reported varying levels of satisfaction with the quality and relevance of questions asked by GPs. It is not always clear to them what kind of advice or answers GPs are seeking. Some hospital specialists also stated that, in some cases, GPs provided them with incomplete or incorrect information, which made clinical decision-making challenging or even impossible (quote 6).

#### Functional Improvements

Most GPs (11/15, 74%) expressed missing the possibility to consult a specialist about patients who are already under specialist treatment. GPs realize that financial aspects play a big part in this problem, but needing to initiate telephone consultation specifically for questions about patient-related issues for patients who are already under specialist treatment instead of having the opportunity to use an e-consultation frustrates GPs. Some GPs admitted to purposely misusing e-consultation for these patients while knowing that the patient was already under specialist treatment.

A chat function or the ability to counterquestion, to discuss about a patient, are suggested by both GPs and hospital specialists (quote 7). This could make an e-consultation more iterative, as some GPs state that this is something they miss in the current state of e-consultation, especially for cases in which important clinical information is missing or the question or response is unclear. Hospital specialists mentioned that in those instances, answering the e-consultation is a time-consuming process as they need to gather information from other resources or write an extensive advice that covers differential scenarios and all possible problems and situations. In contrast, there were also hospital specialists who commented that a chat function would only add to the workload and disrupt their workflow (quote 8).

### Access to Care

#### Ease of Access

Both GPs and hospital specialists recognized the increased accessibility to specialized hospital care through e-consultations (quote 9). Hospital specialists reported that they rarely missed the deadline for e-consultations. In contrast, when referred to an outpatient specialist appointment, wait times could reach up to weeks. GPs and hospital specialists reported on patients appreciating fast access to a hospital specialist (quote 10).

#### Avoidance of Referral

GPs and hospital specialists acknowledge the possibility of an e-consultation to avoid a referral to the hospital (quote 11). This is especially beneficial for older patients, who often face challenges in traveling to the hospital. In addition, patients appreciated avoidance of hospital during the COVID-19 pandemic because they were hesitant to go to the hospital during this time. Outpatient live consultation options remained available for patients who are in need of specialized care. Hospital resources could be directed toward patients who needed specialized care. Some patients explicitly want a hospital specialist’s opinion for reassurance. e-Consultation offers patients this reassurance, where they can get a hospital specialist’s opinion while staying in primary care.

#### Applicability

Hospital specialists noticed that some GPs were struggling with the applicability of e-consultations (quote 12). From their perspective, it is not always clear for GPs in which cases they can or cannot use e-consultations. In some instances, the hospital specialists received questions that they could not answer without physically seeing the patient. They also noticed that e-consultations were not always used in the appropriate situations. In the perspective of hospital specialists, e-consultations are a great medium to ask questions about nonurgent cases, diagnostic testing or results, medical guidelines, and treatment options. However, in case of complex medical problems, high urgency, questions about face-to-face consultations, or follow-up treatment of a patient who is already under care of the hospital specialist, a telephone consultation or physical referral is more appreciated. Hospital specialists suggested that clear user guidelines could help GPs to identify in which cases they can or cannot use e-consultations. Some hospital specialists recommended that flowcharts could make the guidelines clear for GPs.

### Efficiency of Care

#### Preparation

Furthermore, hospital specialists also mentioned that e-consultations provide utility when a referral is still needed. In such cases, e-consultations provide hospital specialists with useful information and enable them to instruct GPs to perform additional diagnostic measurements in preparation for the face-to-face consultation in the hospital (quote 13).

#### Workload

GPs showed disagreement over the workload regarding e-consultation. Most GPs (9/15, 60%) experienced no change in workload, given that the poor accessibility of specialists and the long wait times during a telephone consultation outweigh the extra time it takes to request and process an e-consultation (quote 14). In addition, GPs stated that the number of patients for whom an e-consultation is requested is low to measure a substantial change in workload. In contrast, some said that generating a clinical question, providing relevant clinical information, and implementing the answer of a specialist in patient care cause an increase in workload (quote 15). However, most hospital specialists (11/17, 65%) shared the opinion that e-consultations did not influence their workload.

### Educational Value for GP

#### Overview

GPs and hospital specialists generally praised the educational value of e-consultation (quotes 16 and 17). Hospital specialists were optimistic about the educational abilities that e-consultations provide. Hospital specialists acknowledged that the extent of their knowledge about their own discipline is greater than that of GPs, as GPs have a much broader field to focus on. Therefore, hospital specialists valued that they could exchange their knowledge through e-consultations and assist GPs with any knowledge gaps and diagnostic uncertainties they may have. Hospital specialists explained that when answering a question, they try to substantiate their advice, hoping that GPs will gain knowledge from the provided information. In turn, GPs reported appreciating hospital specialists substantiating their feedback, making it easy for the GP to remember the response when encountering the same problem in a different patient.

#### Training Purposes

Another possible improvement suggested by GPs is to identify gaps in knowledge of GPs by analyzing the asked questions (quote 18). GPs mentioned that hospital specialists may often receive questions from GPs about the same issue and that these topics could be educational training material. Although hospital specialists reported on trying to provide as much feedback as possible to the GP through their answers, they expressed that there were barriers to doing so. A concern they raised was that answers to the e-consultations appear in the medical history and record of the patient. Therefore, patients also have access to any feedback that the hospital specialist provides to the GP through their answers. In addition, hospital specialists cannot receive a response to their answers from GPs owing to the unidirectional nature of e-consultations. Consequently, they receive little to no feedback from GPs.

### GP-Patient Relationship—Strength of Relationship

In general, GPs reported increase in responsibilities over patients for whom an e-consultation is requested. GPs are positive about the ability to keep patients in primary care. GPs reported having questions about liability when implementing e-consultation, but because GPs set strict boundaries and are critical about incomplete or useless responses by specialists, no liability issues have been encountered yet. A few GPs mentioned experiencing improvement in the GP-patient relationship. GPs also report on patients trusting the advice of the GP more when it is backed by a hospital specialist (quote 19).

## Discussion

### Principal Findings

GPs and hospital specialists experience a positive effect of the wide availability of e-consultations with regard to the communication between GP and hospital specialist, access to care, efficiency of care, and educational value of e-consultation for the GP. However, GPs and hospital specialists regretted that their communication became more distant. Differing opinions were expressed regarding the quality of questions and responses, applicability of e-consultation, and effect on the workload, with some GPs and hospital specialists reporting negative experience regarding these aspects, whereas others did not. Possible suggestions were made regarding functional improvements and the use of e-consultations for training purposes.

### Comparison With Existing Literature

In this study, we found a number of similar themes in the perception of both GPs and hospital specialists regarding electronic consultations. These findings are consistent with those of the study conducted by Liddy et al [21]. In the study, 3 focus groups with both primary care providers and hospital specialists were conducted, and similar themes were found compared with those found in our own study. Furthermore, in the study by Liddy et al [21], a number of themes are described that did not emerge in our own study, including improved care for rural and underserved populations and governance.

The GPs and hospital specialists interviewed in our study reported that more efficient communication between GP and hospital specialist, with the ability to take time to formulate a good clinical question, provided with relevant clinical information, makes transmission of the problem clear and more efficient. This is consistent with findings from multiple other studies [19,22-24]. However, they also reported on the communication becoming more distant and acknowledged that dedication from both GP and hospital specialist is needed to formulate clear and high-quality questions and responses and clear guidelines regarding the applicability of e-consultations. Previous studies showed that the completeness of the information and the complexity of the question determined the quality of the response [25,26]. GPs reported improvement in the GP-patient relationship, with patients trusting the advice of the GP more when it is backed by a hospital specialist. GPs also experience increase in responsibilities over patients for whom an e-consultation is requested and are positive about the ability to keep patients in primary care. Consistent with this, previous studies showed that GPs felt more capable of managing the patient within their own practice by using e-consultations [15]. Furthermore, some functional improvements were suggested in the form of a chat function or the ability to send and receive feedback. e-Consultation made specialist care and consultation more accessible for GPs, resulting in patients receiving the needed care quickly and avoiding a referral to the hospital, which is especially useful for older patients or during the COVID-19 pandemic when patients were hesitant to visit the hospital. There are multiple studies reporting on fast access to specialized input [27-45] and avoidance of referrals [12,21,40,46-54]. Furthermore, GPs and hospital specialists reported on increased efficiency of care. Hospital resources were directed toward patients who need specialized care owing to the prevention of unnecessary hospital visits. However, even when the patient was still referred to the hospital specialist, e-consultation was beneficial by providing a way to prepare for this face-to-face visit. GPs and hospital specialists praise the educational value of e-consultation, and some suggest using e-consultations to identify gaps in knowledge to be used for training purposes. These findings are consistent with those of previous studies, as these studies pointed out that GPs frequently identify a gap in clinical knowledge and that these gaps, most likely, are filled by an informal consultation with a specialist [16,23,55-57].

### Strengths and Limitations

This study has several strengths. To date, this is the first study to explore the in-depth views of both GPs and hospital specialists on the availability of e-consultations for medical interprofessional communication. The current COVID-19 health crisis shows the demand for a shift in patient care, where eHealth is becoming the new standard [58]. Different views and perceptions of providers and users of different eHealth services help to further optimize these services. Moreover, interviews were conducted until data saturation was reached according to the estimation by Moser et al [20]. When data saturation was reached, 2 additional interviews were conducted to verify this. In qualitative research, this is methodically the best strategy to determine sample size [59]. Trustworthiness and reliability of the data were ensured by involving 2 coders in the coding process. At the same time, incorporating 2 coders added an additional level of scrutiny and rigor to the coding process, as the different perspectives of both researchers produced a more thorough data analysis. Finally, this study takes the perception of both GPs and hospital specialists into account. Caregivers from different departments were chosen to ensure that a variety of perspectives were included. Similarly, it allowed us to investigate the applicability within different departments. Owing to the semistructured construction of the interviews, they were not restricted to specific questions, which enabled us to redirect the interview in real time. Ultimately, this led to more in-depth answers and uncovered views that would have not been revealed otherwise.

The study also has some limitations. First, the recruited GPs and hospital specialists were experienced in requesting e-consultation and were frequent users of the service. Therefore, we cannot rule out selection bias, and possible negative perceptions of those physicians who had tried e-consultation but did not proceed to use it might be missed. Perceptions of e-consultation can vary depending on user experience and expectation, as has been shown in previous studies [60,61].

### Implications for Clinicians and Policy Makers

Digital interprofessional communication in the form of e-consultations for GPs to consult hospital specialists for nonurgent patient-related questions is regarded as a potential tool to achieve substitution of care, which in turn can reduce health care costs. Through e-consultations, patients can receive care in general practice, which is less costly compared with specialized hospital care [62]. In recent years, health care costs have greatly increased in the Netherlands; in particular, the costs of specialized hospital care are growing at a rapid pace. Previous studies conducted by the Dutch Institute for Health Care Research has shown that substitution of care and reduction of referrals for specialized hospital care can greatly decrease the annual health care costs [62,63]. Furthermore, our analysis shows that e-consultations ensure better access to specialized hospital care and efficiency of care. This is especially important during times when hospital care is already under great demand. Moreover, improved access to specialized hospital care is also important for patients with the greatest health care needs and those who face difficulty in reaching hospitals, such as patients who are frail, older patients, or those who have physical disabilities. In addition, substitution of specialized care by GP-provided care can result in better health care outcomes, as GPs are more likely than hospital specialists to provide continuous and comprehensive care. However, policy makers need to be aware that expanding primary care services and shifting tasks from specialized hospital care to primary care can add to the workload of GPs [5]. e-Consultation is not a one-size-fits-all tool; it has advantages and disadvantages, which should be taken into consideration by a physician when choosing a digital health tool. There will be other tools that are more suitable for certain situations.

In the future, it would be interesting to investigate the link between health literacy and e-consultation, to see whether a patient needs to be (digitally) health literate to be involved in an e-consultation and, subsequently, if the e-consultation could potentially result in an increased gap in health equity. Furthermore, there are legal and ethical questions that arise when looking at e-consultation, for example, about who is responsible if anything goes wrong. These legal and ethical questions need further investigation in the future.

### Conclusions

In conclusion, interviews with GPs and hospital specialists from the Netherlands revealed a number of shared themes regarding the impact of e-consultation on patient care. The GP has a unique gatekeeper role in the Dutch health care system, and e-consultation may play a role in facing the challenges associated with the increased shift from specialized to primary care over the past years. Nonetheless, e-consultation has advantages and disadvantages that should be taken into consideration. In the future, clinicians and policy makers can use the insights obtained from this study to further optimize and implement e-consultation in clinical practice.

## Appendix

Multimedia Appendix 1 Semistructured interview questions.

## Abbreviations

GP: general practitioner
