# Supplementary material for: General Practitioner Use of e-Consultation to Consult Hospital Specialists: Interview Study to Obtain Physician’s Perceptions About Digital Interprofessional Communication
Source: J Med Internet Res. 2023 Mar 28;25:e40318. doi: 10.2196/40318 (PMC10131626; doi:10.2196/40318)
Supplement: Multimedia Appendix 1 [file jmir_v25i1e40318_app1.docx]

*Appendix 1. Semi-structured interview questions*

*Informed consent*

To analyse the data from the interviews, I would like to record the interview (audio only). The interview will be transcribed right after. The recorded interviews will be stored safely. During transcription the interviews will be anonymized. Personal data will not be mentioned in the article. Do you give consent to recording this interview?
- Yes
- No

Do you have any further questions before we start this interview? Do you agree on starting this interview?
- Yes
- No

The interview

1. General characteristics
a. Can you tell me some general characteristics about yourself?
i. What is your age?
ii. What is your specialization? (SCP only)
iii. How long have you been working as a GP or SCP?
iv. How many GPs work in your practice? (GP only)

2. E-consult experiences
a. How did you learn about the e-consultation?
b. How many e-consultations have you (approximately) requested or answered?
c. How much time do you on average spend on writing or answering an e-consultation?
d. What are your views on the e-consultation?
i. What is positive about the e-consultation?
ii. What would you like to see differently?
iii. Are there any logistic problems that you encountered during requesting or answering an e-consultation?
iv. Did the threshold for consulting an SCP lower because of the e-consultation? (GP only)

e. In which case(s) do you think the e-consultation is a good alternative for a referral?
f. What do you think is the effect of the e-consultation on the quality of care?
g. What is the view of the patient on the e-consultation? (GP only)
i. How do you inform the patient about this? How do they react? (GP only)

3. Content e-consultation
a. What do you think about the quality of the questions or responses?
i. When the question or response is incomplete in your view > what kind of information do you miss?
ii. If the GP or SCP states a question or response is unclear > what do you do when a question or response is unclear?
iii. What do you do if you cannot answer a question? (SCP only)
iv. If the GP or SCP states that the quality of question or response was good > what makes it good? Or bad?
b. For which kind of cases is the e-consultation well suited or not suited?
i. In which situations would you rather discuss the case by phone?
c. What do you think about the educational value of the e-consultation?

4. Collaboration and communication between GP and SCP
a. What is the effect of the e-consultation on the collaboration between the GP and SCP?
b. Can you describe the communication between you and GP/SCP regarding the e-consultation?
i. What would you think about a chat functionality?
c. How does the e-consultation influence your work load?
i. What do you think about the 48 hour deadline? Do you think this is manageable or do you need more time? (SCP only)
ii. Did the GP-patient relationship change because of the e-consultation? How? (GP only)

5. Applicability e-consultation
a. What do you think about the applicability of the e-consultation within your specialism? (SCP only)
b. What do you look at when answering an e-consultation? (SCP only)
c. What do you think is clinically relevant information for an e-consultation?

6. Feedback (from and to GP)
a. What would you think about the ability to give and receive feedback?

i. How would you like to receive feedback?
b. What would you think about the possibility of requesting an e-consultation yourself as a SCP? For example asking a GP a question about a patient who was (recently) admitted to the hospital or visited the outpatient clinic. (SCP only)
c. What do you think are the most important points of feedback for GPs or SCPs regarding e- consultations?

7. Wrap-up
This is the end of the interview.
Are there any subjects you would like to talk about, which were not talked about during the interview?
Would you like to receive the results of our study?
